# Assessing Tn5 and Sleeping Beauty for transpositional transgenesis by cytoplasmic injection into bovine and ovine zygotes

**DOI:** 10.1371/journal.pone.0174025

**Published:** 2017-03-16

**Authors:** R. J. Bevacqua, R. Fernandez-Martin, N. G. Canel, A. Gibbons, D. Texeira, F. Lange, G. Vans Landschoot, V. Savy, O. Briski, M. I. Hiriart, E. Grueso, Z. Ivics, O. Taboga, W. A. Kues, S. Ferraris, D. F. Salamone

**Affiliations:** 1 Animal Biotechnology Laboratory, Facultad de Agronomia. INPA-CONICET, Buenos Aires University, Buenos Aires, Argentina; 2 Experimental Station Bariloche, INTA, Bariloche, Argentina; 3 Laboratorio de Fisiologia e Controle da Reprodução, FAVET, UECE, Ceará State, Brasil; 4 Cloning and Transgenesis Laboratory, Maimónides University, Buenos Aires, Argentina; 5 Paul-Ehrlich-Institute, Langen, Germany; 6 CICVyA Biotechnology Institute, INTA Castelar, Buenos Aires, Argentina; 7 Friedrich-Loeffler-Institut, Neustadt, Germany; University of Connecticut, UNITED STATES

## Abstract

Transgenic domestic animals represent an alternative to bioreactors for large-scale production of biopharmaceuticals and could also provide more accurate biomedical models than rodents. However, their generation remains inefficient. Recently, DNA transposons allowed improved transgenesis efficiencies in mice and pigs. In this work, Tn5 and Sleeping Beauty (SB) transposon systems were evaluated for transgenesis by simple cytoplasmic injection in livestock zygotes. In the case of Tn5, the transposome complex of transposon nucleic acid and Tn5 protein was injected. In the case of SB, the supercoiled plasmids encoding a transposon and the SB transposase were co-injected. *In vitro* produced bovine zygotes were used to establish the cytoplasmic injection conditions. The *in vitro* cultured blastocysts were evaluated for reporter gene expression and genotyped. Subsequently, both transposon systems were injected in seasonally available ovine zygotes, employing transposons carrying the recombinant human factor IX driven by the beta-lactoglobulin promoter. The Tn5 approach did not result in transgenic lambs. In contrast, the Sleeping Beauty injection resulted in 2 lambs (29%) carrying the transgene. Both animals exhibited cellular mosaicism of the transgene. The extraembryonic tissues (placenta or umbilical cord) of three additional animals were also transgenic. These results show that transpositional transgenesis by cytoplasmic injection of SB transposon components can be applied for the production of transgenic lambs of pharmaceutical interest.

## Introduction

Transgenic animals constitute an important tool for basic and applied research. In particular, transgenic farm animals could provide more accurate biomedical models than rodents [1,2] and also represent an alternative to current bioreactors for large-scale production of biopharmaceuticals [3,4]. The feasibility of using domestic species for the generation of highly complex proteins, which cannot be efficiently manufactured by conventional microorganisms or animal cell bioreactors, has been demonstrated for the past two decades [4–9]. However, the techniques available for transgenic animals production remain inefficient.

Pronuclear microinjection and blastocyst injection with embryonic stem cells (ES) are the techniques most commonly used to produce transgenic mice [10,11]. However, their application to domestic species requires proper pronuclei visualization and species-specific ES cells maintenance, respectively, which up to date are not efficiently achieved in livestock [12,13]. For these reasons, somatic cell nuclear transfer (SCNT) soon turned into the technique of choice for the production of transgenic domestic species, and it has allowed the production of transgenic cattle [4;14], sheep [3] and pigs [15]. Nevertheless, the efficiency of SCNT remains low [16,17], and only a fraction of the transgenic offspring produced by any of these methods shows the desired transgene expression. This is mainly consequence of the random transgene integration as concatamers that can lead to epigenetic silencing of the transgene [18–20].

In recent years, active transgenesis approaches were introduced, based on the use of enzymes that actively promote the integration of the transgene, in opposition to traditional transgenesis techniques that rely on the introduction of transgenes into existing double stranded breaks in the genome. One of the first active transgenesis techniques introduced was lentivirus-mediated transgenesis [21]. Despite its high efficiency, this technique shows many drawbacks including high embryo lethality rates, relatively small cargo capacity of the vector (9.5 kb) and biosafety concerns related to virus. A more promising alternative for transgenic animals production is transpositional transgenesis [22–25]. The ability of DNA transposons to move from one genomic location to another by a cut-and-paste mechanism was soon adapted as a molecular biology tool. Several transposition systems were identified and hyperactive transposase versions were isolated [26,27].

Tn5 is one of the best-characterized bacterial transposon systems; however, only one report has been published using hyperactive Tn5 mutant transposase (*Tn5) in combination with intracytoplasmatic sperm injection (ICSI) for transgenesis in mice [26]. The *Tn5 hyperactive transposase protein is commercially available (Epicentre TNP10622). This protein forms DNA:transposase complexes, called transposomes, in the absence of Mg^+2^, by specific binding to 19-bp recognition Mosaic End (ME) sequences of the transposon [28,29]. Once inside of the cell, the transposase becomes activated by the cellular Mg^+2^ levels and integrates the transposon DNA into a random position favoring GATC(A/T)GATC in bacterial chromosomes [30–33].

Almost twenty years ago, the molecular reconstruction of Sleeping Beauty (SB) transposon from salmonid genomes [34] expanded the utility of transposon-based technologies to vertebrates. In 2009, Mátés et al. [27] reported the efficient production of transgenic mice by pronuclear injection of the hyperactive version of the SB transposase (SB100X). More recently, transgenic pigs and cattle were produced by simple cytoplasmic injection of the hyperactive SB100X system with reporter transposons [25,35] and also by cloning using somatic cells modified with SB100X [23,24].

In previous reports, we showed that it was possible to produce transgene-expressing embryos by simple cytoplasmic injection of transgenes into IVF presumptive zygotes [36,37]. In addition, we observed that cytoplasmic injection with I-*Sce*I meganuclease digestion reaction into bovine IVF zygotes resulted in increased number of transgene signals in embryos, detected through fluorescence *in situ* hybridization [38]. In this work, we evaluated Tn5* and SB100X active transgenesis mediated by direct cytoplasmic injection. As genome activation in bovine embryos takes place at day 4 [39], we speculated that injection with the Tn5 transposome complex would favor earlier transgene integration. However, the high activity levels reported for hyperactive SB100X also seemed promising for direct cytoplasmic plasmid injection. For that reason, both systems were evaluated *in vitro* in bovine zygotes with a reporter gene and *in vivo* in ovine zygotes with recombinant human coagulation factor IX (rhFIX) driven by a beta-lactoglobulin promoter.

Currently, therapies for hemophilia B are based on protein infusion treatments using recombinant proteins, or components derived from human blood plasma. The production of recombinant human factor IX in sheep milk would provide an alternative source of factor IX at a lower cost, but also free from potential infectious risks associated with products derived from human blood. Transgenic sheep [3] and goats [40] capable of producing recombinant human factor IX in milk were previously produced by somatic cell nuclear transfer. In this work, we demonstrate that transgenic farm animals of pharmaceutical interest can also be produced by simple cytoplasmic injection of plasmids coding for both components of SB transposition system into zygotes with a high efficiency.

## Materials and methods

### Chemicals

Except where otherwise indicated, all chemicals were obtained from Sigma Chemical Company (St. Louis, MO, USA).

### Animal welfare

The *in vivo* experiments in sheep were in accordance with the recommendations of the guidelines stated in the Guide for the Care and Use of Agricultural Animals in Agricultural Research and Teaching. The study design was approved by the Institutional Committee of Use and Welfare of Experimental Animals, under number 6/13. Sheep were housed at Maimonides University animal facilities, and transgenesis experimentation was done after approval of the Agricultural Biotechnology Assessor National Committee (CONABIA), resolution 533168–13. Sheep were provided daily care and feeding, with permanent ad-libitum access to water.

### Generation of plasmid vectors

**Fig 1:**
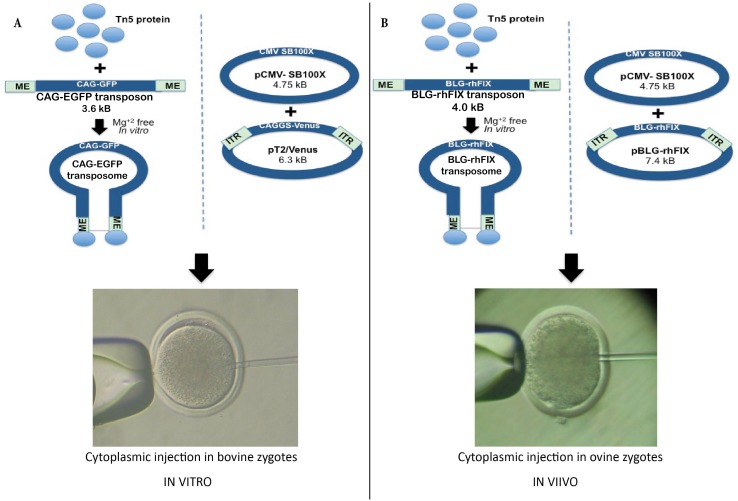
Tn5 and Sleeping Beauty Transpositional Transgenesis in bovine and ovine. A) Schematic depiction of the plasmids used for *in vitro* transposition with reporter transposons in bovine zygotes. B) Schematic depiction of the plasmids used for *in vivo* transposition with rhFIX transposons in ovine zygotes. The Tn5 protein was complexed to the transposon *in vitro*, in Mg^+2^ free medium. The resulting transposome was used for injection into presumptive zygotes. The mixture of SB plasmids was deposited directly into the cytoplasm of the zygotes.

The plasmid pCX-EGFP, expressing EGFP under the control of the CAG promoter, was a kind gift from Dr. Masaru Okabe [41]. For Tn5 *in vitro* experiments in bovine, pMOD-2/CX-EGFP plasmid was created by cloning a 2994 bp pCX-EGFP *Sal*I/*Hin*dIII fragment containing the *egfp* gene, the CAG promoter and the Rabbit beta-globin polyA into the *Sal*I/*Hin*dIII sites present in the MCS of EZ-Tn5™pMOD™-2 (Cat. No. TNP10622; Epicentre). Then, the transposon flanked by its ME sequences, was excised from pMOD-2/CX-EGFP by digestion with *Pvu*II, and the 3.6 Kb fragment containing the transposon was separated in a 1% agarose gel, purified with a Gel extraction kit (Cat. No. 28704; Promega) and used for transposome assembly (Fig 1). For SB bovine *in vitro* experiments, pT2/Venus (6.3 kB) and pCMV-SB100X (4.75 kB) were used, as previously described [27]. For ovine *in vivo* experiments, a 3435 bp DNA sequence containing B-lactoglobulin minimal promoter, bovine beta casein signal peptide, human factor IX cDNA and rabbit beta globin polyA was *in vitro* synthesized and cloned into pUC57 (pUC57-rhFIX) by Genscript (NJ, USA). For Tn5 transposon, the pUC57-rhFIX *Eco*RI-*Hin*dIII fragment was cloned into the MCS of EZ-Tn5™pMOD™-2. The 4.0 kB rhFIX-Tn5 transposon was PCR amplified and used for transposome complex formation according to the manufacturer recommendations.

For the rhFIX SB transposon, the pUC57-rhFIX *Xba*I/*Bam*HI fragment was cloned into the pT2 vector, which was obtained as a 3953 bp *Nhe*I/*Aat*II fragment from pT2/Venus [27]. Previous to ligation, both fragments were treated with T4 polymerase to obtain blunt ends. The correct sequence of the plasmid was confirmed by Sanger sequencing with optimized fluorescent terminator protocols (Genomic Unit, Biotechnology Institute, INTA Castelar). The resulting transposon plasmid (7.4 kB) was named pT2/rhFIX. A schematic depiction of the plasmids employed is included in Fig 1.

### *In vitro* production of bovine zygotes

Bovine ovaries were collected from slaughterhouses and transported to the laboratory at 25–30°C. Oocytes covered with at least 3 layers of granulosa cells were selected for *in vitro* maturation (IVM), as previously reported [42]. Briefly, the maturation medium was bicarbonate-buffered TCM-199 (31100–035; Gibco, Grand Island, NY, USA), containing 10% fetal bovine serum (FBS, 013/07; Internegocios, Buenos Aires, Argentina), 10 μg/ml follicle stimulating hormone (NIH-FSH-P1, Folltropin®, Bioniche, Caufield Junction Caufield North, Victoria, Australia), 0.3 mM sodium pyruvate (P2256), 100 μM cysteamine (M9768), and 2% antibiotic-antimycotic (ATB, 15240–096; Gibco). The oocytes were incubated for 22 h under mineral oil (M8410) in 100 μL droplets, under 6.5% CO_2_ in humidified air at 39°C. For IVF, frozen semen was thawed in a 37°C water bath for 30 s. Spermatozoa were then centrifuged twice (490g x 5 min) in Brackett-Oliphant medium (BO) [43] and resuspended in BO supplemented with 5 mM caffeine (C4144) and 20 IU/ml heparin (H3149). Spermatozoa were adjusted to 30 x10^6^/ml and diluted to half concentration (15 x10^6^/ml) with BO containing 10 mg/ml fatty acid-free bovine serum albumin (A6003). COCs were washed twice with BO medium plus 5 mg/ml FAF-BSA and subsequently exposed to the sperm suspension for 5 h in a 100μl droplet at 39°C under 5% CO2 in humidified air. After IVF, cumulus cells were removed from presumptive zygotes by vortex for 2 min in hyaluronidase (H-4272) (1 mg/ml in Dulbecco´s phosphate buffered saline- PBS). Then, presumptive zygotes were washed in Hepes-TALP, selected by visualization of at least one polar body, and immediately used for microinjection.

### Preparation of Tn5 transposome complex, ccc-SB plasmids and cytoplasmic injection

For Tn5 transposition, the 8 μl reaction solution was prepared by mixing together 2 μl of 200 ng/ul *egfp* or rhFIX transposon (Tnsp) in TE buffer (10 mMTris-HCl, pH 7.5, 1 mM EDTA), 4 μl Tn5 (1U/ll; Cat. No TNP10622; Epicentre) and 2 μl glycerol. The reaction was mixed by vortexing and incubated for 30 min at room temperature. The transposon fragment at 16 ng/μl was injected either as transposome, (ME)*egfp*:Tn5 or (ME)rhFIX:Tn5 Tnsp:Tn5 complex, or as transposon alone, (ME)*egfp* or (ME)rhFIX, as control. For SB transposition, a transgene mixture consisting of 10 ng/μl pT2/Venus or 10 ng/μl pT2/hFIX and 5 ng/μl pCMV-SB100X was prepared. As controls, transposon or transposase alone were injected. *In vitro* fertilized bovine oocytes or ovine presumptive zygotes recovered from superovulated ewes were transferred to 20 μl droplets of Hepes-TALP and cytoplasmically injected, using a 9 μm pipette, with the transposon mixtures in 10% PVP in a volume <10 pl. The PVP used for microinjection was Mg^+2^-free to prevent the premature activation of the Tn5 transposase. Presumptive zygotes were subsequently cultured or transferred to surrogate animals as described below.

### *In vitro* embryo culture

Presumptive zygotes were cultured in 50 μl droplets of synthetic oviduct fluid (SOF) medium [44] supplemented with 2.5% FBS at 39°C in 6.5% CO_2_ in humidified air. The embryos were transferred to a new droplet every 48 h. Cleavage was evaluated at day 2 and the number of blastocysts at day 7.

### Evaluation of EGFP/ Venus fluorescence in embryos

All the *in vitro* produced embryos were briefly exposed to blue light using an excitation-filter at 488 nm and an emission-filter at 530 nm to determine *egfp* and *Venus* gene expression. Injected embryos were evaluated at days 5 and 7 post-IVF.

### Southern blotting

For embryo Southern blot, whole genome amplification (WGA) was performed on individual embryos using the REPLI-g Midi Kit (Qiagen, Valencia, CA) according to the ‘‘Amplification of Blood or Cells” protocol detailed within the product manual. Approximately 10 μg of resulting DNA was digested with *BamH*I, *Cla*I and *Sal*I in *BamH*I buffer with two-fold excess of *Cla*I and *Sal*I at 37°C overnight, and afterwards it was resolved in 1% agarose gel and transferred to positively charged nylon membranes using standard methods. Membranes were pre-hybridized and then hybridized at 65°C in Church buffer [45]. As a probe, a *Pvu*II-*Pvu*II *egfp* fragment from Tn5 transposon was labeled by nick translation with α-32P CTP (Prime-a-Gene Labeling System Promega, U1100). As positive controls, 10 pg and 1 ng *Sal*I-*Xba*I pCX-EGFP 1.6 Kb fragment were included.

Animal Southern blot was performed under similar conditions than embryo Southern blot. In this case, the DNA was extracted from blood and tissues using NucleoSpin® Blood L or Tissue kit (Macherey Nagel 740954.20 and 740952.50). Afterwards, DNA was digested with *Pvu*II at 37°C overnight. As a probe, a 354 bp PCR fragment obtained from pT2/rhFIX (forward primer: atatgctggctgccatgaac; reverse primer: gcttctcgagataacttcgtataatg) was labeled as before. The membranes were cross-linked and exposed overnight. They were resolved using a Typhoon FLA 9000 (GE Healthcare Life Sciences).

### Superovulation and laparoscopic artificial insemination of donor ewes

Sixteen multiparous Hampshire Down sheep, 4 years old, in good body condition (>3 out of 5; scale from 0, emaciated, to 5, obese) and moderate body weight (55.8 ± 2.2 kg) were used during the breeding season. The estrous cycle was synchronized by the insertion of an impregnated intravaginal progestagen pessary (60 mg of medroxyprogesterone acetate, Progespon®, Syntex, Argentina) for 14 days. The superovulatory treatment consisted of i.m. injection of a total of 200 mg of pFSH (Folltropin-V^®^, Bioniche, Canada) in six decreasing doses (50 mg x 2, 30 mg x 2, 20 mg x 2) applied twice daily, from the morning of day 12, 48 h before pessary removal, to 12 h after sponge withdrawal. A single i.m. dose of eCG (200 IU, Novormon 5000®, Syntex, Buenos Aires, Argentina) was administered at the time of progestagen removal at day 14 [46]. Laparoscopic insemination was performed 60 h later under general anesthesia, consisting of a pre-treatment with 20 mg/ml xilacina (Xilacina 20, Richmond Vet Pharma, Argentina), followed by 100 mg propofol (PropoVet, Richmond Vet Pharma, Argentina), 15 mg midazolam (Midazolam, Richmond Vet Pharma, Argentina), 15 mg isoflurano (Isoflurano Baxter, Baxter Healthcare, USA) and 20 mg tramadol hydrochloride (Algen 20, Richmond Vet Pharma, Argentina). Ewes were artificially inseminated with 100 million frozen-thawed spermatozoa doses, from the same batch and ram, by the laparoscopic method described by Maxwell and Butler [47].

### Recovery, microinjection and transference of presumptive zygotes

Donor females were deprived of food and water for 12 h prior to surgical recovery. Sixteen hours after the laparoscopic insemination, presumptive zygotes were recovered by laparotomy under general anesthesia, as described for laparoscopic insemination. Briefly, oocytes and zygotes were flushed through the oviducts using 38°C pre-warmed phosphate-buffered saline (PBS) supplemented with 1% FBS in a retrograde fashion. This involved injection with 5 ml of stocking medium (Syngro®, Bioniche, USA) (per each oviduct) with a sterile syringe fitted with a needle (18 g), from the utero tubal junction towards the fimbria, and liquid collection in a sterile plastic tube. Immediately after recovery, the presumptive zygotes were cytoplasmically microinjected with Tn5 or SB transposition systems. In both cases, recombinant human Factor IX under beta-lactoglobulin promoter cDNA transposon was employed. The injected presumptive zygotes were placed in stocking medium until transfer (3 h post-recovery).

Approximately 3 h after microinjection, the zygotes were transferred into the oviduct of synchronized estrous recipients (2 to 4 zygotes per ewe). Briefly, 22 adult Hampshire Down recipients were synchronized using impregnated intravaginal progestagen pessary (60 mg medroxyprogesterone acetate, Progespon®, Syntex, Buenos Aires, Argentina) for 14 days. On day 14, sponges were removed and a single i.m. dose of 200 IU of eCG (Novormon 5000®, Syntex, Buenos Aires, Argentina) was administered. Progestagen pessary removal took place 48 h earlier in recipient ewes than in donors. The recipient females were deprived of food and water for 12 h prior to surgical transfer. The presumptive zygotes were transferred under general anesthesia (as previously described), in 0.2 ml of stocking medium and using a Tomcat catheter (plastic device). The microinjected zygotes were expelled with the aid of a 1-ml syringe. The zygotes were deposited in the upper third of the ipsilateral oviduct of the ovary showing a normal corpus luteum. Pregnancy and embryo survival rates were recorded 28 days after transfer, using trans rectal ultrasonography, with a 5MHz linear array transducer (Mylab 30, Esaote SpA).

### Transgene detection in animal tissues by PCR

DNA was extracted from blood using NucleoSpin® Blood L kit (Macherey Nagel 740954.20) and from skin of newborns using NucleoSpin® tissue kit (Macherey Nagel, 740952.50). Post mortem, DNA was extracted from additional tissues: lung, muscle, kidney and liver. The PCR and nested PCR were performed with GoTaq® DNA polymerase (Promega, M8291). Both PCRs had the same cycling conditions: 1 cycle at 95°C for 2 minutes, followed by 95°C for 20 seconds, 58°C for 20 seconds and 72°C for 45 seconds, during 40 cycles, and a final extension at 72°C for 5 minutes. The first PCR product size (oFIXter3F, GGGTGAAGAGTGTGCAATGA—oFIXter4R, GGCTTCATGATGTCCCCATA) was 222 bp and the nested one (oFIXter1F, TCCCGGTATGTCAACTGGAT -oFIXter2R, TTTTGGCAGAGGGAAAAAGA) was 151 bp. To assess possible integration of pCMV-SB100X, a second PCR was performed on the transgenic animal tissues using the PCR conditions previously described and primers oSB100-1F (aagaagccactgctccaaaa) and oSB100-2R (ttcctcctgacagagctggt). As positive control, 10 fg of pCMV-SB100X plasmid were used. The expected amplicon was 567 bp.

### Identification of integration sites by splinkerette PCR

Transposon-genomic DNA junctions were determined using splinkerette PCR as described previously [48]. The purified PCR product was cloned into the pGEM-Teasy vector (Promega, Madison, USA), and the DNA sequence was determined by standard sequencing technology (ABI3730XL Applied Biosystems, Foster City, California). Sequences were analyzed with BLAST software (www.ensembl.org) [49]. The integration site was confirmed by PCR and nested PCR, performed with GoTaq® DNA polymerase (Promega, M8291) and the same cycling conditions for both PCRs: 1 cycle at 95°C for 2 minutes, followed by 95°C for 10 seconds, 58°C for 10 seconds and 72°C for 20 seconds, during 40 cycles, and a final extension at 72°C for 5 minutes. The first PCR was performed with primers ointtransp1F (AAGCAGGTTCCCTGTGAAAA) and ointtransp1R (CCAAGCTGTTTAAAGGCACA), resulting in a 475 bp product. For the nested PCR, primers ointtransp2F (CAAGCACCAACACAGAAAGG) and ointtransp2R (TCTGACCCACTGGAATTGTG) were employed, resulting in a 282 pb amplicon.

### Statistical analysis

For statistical analyses, the SAS program [50] was used. *In vitro* embryo development and fluorescent expression were compared by Fisher’s exact test analysis. Differences were considered to be significant at P < 0.05.

## Results

### Evaluation of Tn5 and Sleeping Beauty transpositional transgenesis *in vitro* in bovine embryos

**Fig 2:**
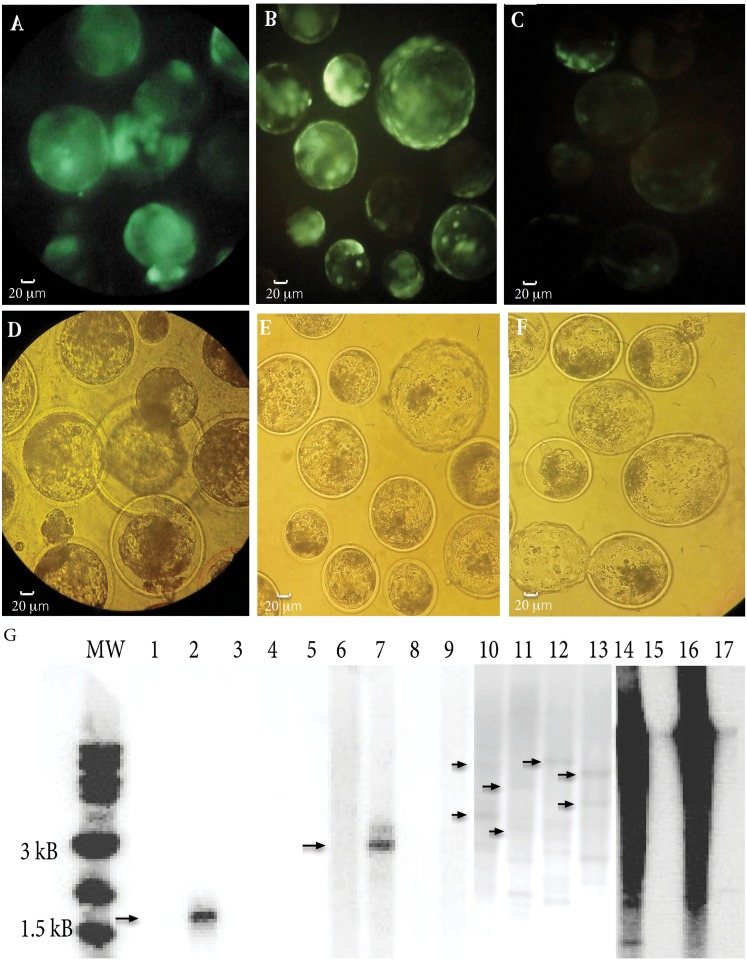
Tn5 and Sleeping Beauty transpositional transgenesis by direct cytoplasmic injection in bovine zygotes. A,D) Blastocysts produced by injection with (ME)*egfp*:Tn5 transposome. B,E) Blastocysts produced by injection with pT2/Venus and pCMV-SB100X. C,F) Blastocysts produced by injection with pT2/Venus alone. A-C) Under bright light. D-F) Under blue light (488 nm). G) Embryo Southern blot performed on DNA amplified from Tn5 and Sleeping Beauty system injected blastocysts. WGA was performed to obtain enough DNA for Southern blot. Lane 1: 10 pg *Sal*I-*Xba*I pCX-EGFP 1.6 Kb positive control. Lane 2: 1 ng *Sal*I-*Xba*I pCX-EGFP 1.6 Kb positive control. Lane 3,5: Empty lanes. Lane 4: IVF blastocyst (negative control). Lane 6–9: Tn5 transposon control. Lane 10–13: Tn5 transpositional transgenesis blastocysts. Lane 14–17: Sleeping Beauty transpositional transgenesis blastocysts. MW: NEB 1 kB Ladder of molecular weight.

Both Tn5 and Sleeping Beauty system were evaluated for their suitability to mediate transgenesis after cytoplasmic injection into bovine preimplantatation embryos. A schematic depiction of the procedure is included in Fig 1A. Results for Tn5 transgenesis are summarized in Table 1. No statistical differences were detected among injection with the transposon alone or complexed with Tn5 in terms of *egfp* expression (Fig 2A and 2D). The injection with the transposon alone or with the transposome complex resulted in lower blastocyst rates than observed for the IVF control (p<0.05).

**Table 1:** *In vitro* embryo development and *egfp* expression rates for Tn5 transpositional transgenesis in bovine.

Treatment	n	Cleavage (%)	Blastocysts (%)	*Egfp+*D5 (%)	*Egfp+* blastocysts (%)*
(ME)egfp:Tn5	1145	131 (90)	73 (50)^a^	86 (59)	65 (89)
(ME)egfp	129	117 (90)	65 (50)^a^	87 (67)	57 (78)
Control	171	163 (95)	107 (63)^b^	*n*.*a*.	*n*.*a*.

^a^ different superscripts in the same column indicate significant differences among treatments (Fisher test; p<0.05).

^b^ different superscripts in the same column indicate significant differences among treatments (Fisher test; p<0.05).

*n*.*a*. non-applicable. (ME)*egfp*: *egfp* transposon. *Egfp* +D5: *egfp* expressing embryos at day 5. The cleavage, blastocyst and *egfp*+ D5 rates were calculated over the total n.

* The percentage of *egfp* blastocysts was calculated over total number of blastocysts.

Results for Sleeping Beauty transgenesis are summarized in Table 2. No statistical differences were detected among groups in terms of development or percentage of embryos expressing *Venus*. However, *Venus* intensity in embryos co-injected with pCMV-SB100X and pT2/Venus was markedly higher respect to those injected with pT2/Venus alone (Fig 2B, 2C, 2E and 2F).

**Table 2:** *In vitro* development and *Venus* expression rates for Sleeping Beauty transpositional transgenesis in bovine.

Treatment	n	Cleavage (%)	Blastocysts (%)	*Venus*+ D5 (%)	*Venus*+ blastocysts (%)*
pT2/Venus-pCMV-SB100X	136	113 (83.0)	58 (42.6)	86 (63.2)^a^	42 (72.4)^a^
pT2/Venus	152	126 (82.9)	54 (35.5)	95 (62.5)^a^	39 (72.2)^a^
pCMV-SB100X	107	98 (91.6)	44 (41.1)	0 (0.0)^b^	0 (0.0)^b^
Control	139	120 (86.3)	62 (44.6)	*n*.*a*.	*n*.*a*.

^a^ different superscripts in the same column indicate significant differences among treatments (Fisher test; p<0.05).

^b^ different superscripts in the same column indicate significant differences among treatments (Fisher test; p<0.05).

pT2/Venus: *Venus* transposon; pCMV-SB100X: Sleeping Beauty transposase. *Egfp* +D5: *egfp* expressing embryos at day 5. *n*.*a*. non-applicable. The cleavage, blastocyst and *Venus*+ D5 rates were calculated over the total n.

* The percentage of *Venus* blastocysts was calculated over total number of blastocysts.

Embryo Southern blot was performed in order to obtain integration evidences. To this aim, whole embryo genome was amplified, because DNA from a single blastocyst is below the Southern blot detection limit. The use of Phi29 (Φ29) polymerase allowed the isothermal generation of DNA fragments up to 100 kb without sequence bias. Blastocysts derived from three treatments were analyzed: non-injected control, non-transposase control (injected with Tn5 transposon fragment alone) and transposon/transposase groups. No signal was detected in the non-injected embryo control and in three out of four blastocysts of the non-transposase control. In the forth blastocyst of this group, a strong band was detected (3.1 kb), that correlates to the digestion of many transposons re-circularized in an episomal structure, probably as concatamers. For the blastocysts injected with (ME)egfp:Tn5 transposome, discrete bands compatible with integrations were detected. Bands of higher molecular weight than the transposon (3.1 kb) were detected in all analyzed embryos in this group. Moreover, smaller bands were also found. These bands could correspond to hybridization with 29 nt at the 5’ end of the probe, opposite to the *Cla*I restriction site. The Southern blot analysis suggests that in Tn5 injected embryos one to three insertions occurred.

The Southern blot analysis of SB-injected embryos produced much higher signal intensities, probably due to persistence of high amounts of episomal (circular) plasmid at the blastocyst stage. In contrast, the halve-life of (ME)egfp:Tn5 transposome appeared to be much shorter (Fig 2G).

### Evaluation of Tn5 and Sleeping Beauty transpositional transgenesis *in vivo* in ovine

In a subsequent step, both transposition systems were assessed *in vivo* for the production of transgenic lambs. In this case, a functional transposon coding for *rh*FIX driven by the minimal beta-lactoglobulin promoter was employed. A schematic depiction of the plasmids is included in Fig 1B. In total, 16 donor ewes were employed and 171 presumptive zygotes were recovered. In average, 10.5±7.1 presumptive zygotes were recovered from 14.3±7.2 corpuses luteal in each donor ewe (74.1% recovery efficiency). A total of 72 presumptive zygotes were finally transferred to 22 recipient animals. As a result, eight pregnancies were established and a transgenic lamb was obtained, showing transgene presence in almost all tissues analyzed. This animal was obtained by Sleeping Beauty transgenesis. Results are summarized on Table 3.

**Table 3:** Summary of Tn5 and Sleeping Beauty transpositional transgenesis *in vivo* in ovine.

Treatment	Rep.	N° injected zygotes	N° transferred zygotes	N° pregnant/ recipients	N° fetuses (tz) [r]	N° born sheep (tz) [r]	N° tg born sheep (tz) [r] {b}
ME(rhFIX):Tn5	1	15	12	2/3	2 (16) [66.6]	1 (8.3)[33.3]	0 (0) [0] {0}
	2	9	9	0/2	0 (0)	0 (0)	0 (0) [0] {0}
	Total	24	21^a^	2/5	2 (9.5) [40]	1 (4.7) [20]	0 (0) [0] {0}
pT2/rhFIX-pCMV-SB100X	1	62	21	3/7	4 (19) [57]	4 (19) [57]	1*(4.7)[14.2]{25}
	2	45	12	1/4	1 (8.3)[25]	1 (8.3)[25]	1 (8.3)[25]{100}
	3	34	18	2/6	2 (11.1)[33.3]	2 (11.1)[33.3]	0 (0) [0] {0}
	Total	141	51^b^	6/17	7 (13.7)[41.1]	7 (13.7)[41.1]	2 (3.9)[11.7]{28.6}

^a^ different superscripts in the same column indicate significant differences among treatments (Fisher test; p<0.05).

^b^ different superscripts in the same column indicate significant differences among treatments (Fisher test; p<0.05).

ME(rhFIX):Tn5: rhFIX Tn5 transposome. pT2/rhFIX-pCMV-SB100X: rhFIX transposon/ Sleeping Beauty transposase plasmids. Rep.: repetition. Tg: transgenic. The rate of fetuses, born sheep and transgenic born sheep were calculated over number of transferred zygotes (tz), and over number of recipient ewes [r]. The rate of transgenic born sheep was also calculated over the total number of born animals{b}.

*the transgene was only detected in liver and placenta.

For Tn5 transgenesis, 21 presumptive zygotes were transferred to 5 recipient sheep, and 2 of them became pregnant. Although one of these sheep was pregnant of siblings, only one of the fetuses proceeded to term. Genomic DNA from skin, placenta and blood from this lamb was genotyped by PCR, but the *rh*FIX gene was not detected. One of the three surrogates that did not get pregnant was transferred with six 2 cell-embryos, cleaved by the time of recovery and therefore, injected on both blastomeres.

**Fig 3:**
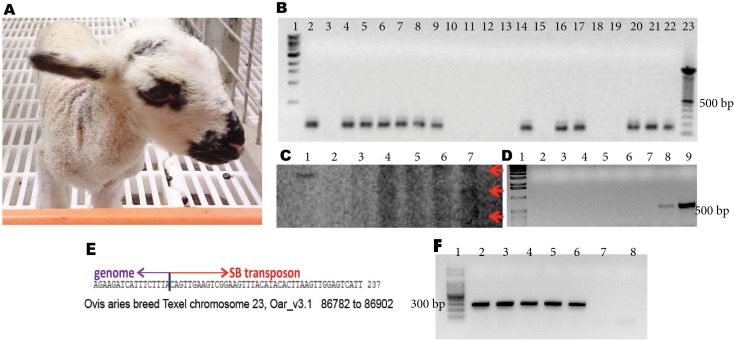
Molecular analysis of transgenic lamb obtained by Sleeping Beauty transpositional transgenesis. A) Transgenic lamb produced by Sleeping Beauty transgenesis. B) Nested PCR specific amplification of the transgene: 1: 1 Kb MW, 2–18: DNA extracted from: 2–7: different skin samples, 8: cryopreserved testis cells, 9: cryopreserved cultured cells derived from umbilical cord, 10–13: cells cultured from different tissues (10: muscle, 11: kidney, 12: lung, 13: placenta), 14: kidney, 15: lung, 16: muscle, 17: placenta, 18: wild type sheep, 19: negative control mix, 20: positive equimolar control, 21: positive control diluted 1/10, 22: positive control diluted 1/100, 23: 100 bp MW. C) Southern blot performed on DNA extracted from different tissues of the transgenic animal: 1: 3889 bp positive control signal, 2: empty lane, 3: DNA from wild type sheep, 4–7: DNA extracted from tissues of transgenic lamb, 4: kidney, 5: lung, 6: muscle, 7: placenta. D) PCR specific amplification of pCMV-SB100X. 1:1 Kb MW, 2–5: 10 ng DNA from kidney, lung, muscle and placenta of transgenic lamb respectively, 6: wild type sheep DNA, 7: negative control mix, 8: positive control diluted 1/10, 9: positive equimolar control. E) The transposon-genomic DNA junctions were determined using splinkerette PCR and standard sequencing technology. The integration site was mapped to ovine chromosome 23, at the 3’ of olfactory receptor 5D18-like by ensemble and BLAST. F) Nested PCR confirmation of the integration site detected by splinkerette PCR, using forward primers on the 5D18-like receptor region and reverse primers on the transposon. Lane 1: MW, lanes 2–4: different skin samples, lane 5: cryopreserved testis cells, lane 6: placenta, lane 7: wild type and lane 8: negative control mix.

For SB transgenesis, 12 donor sheep were employed. Only one-cell embryos were transferred into 17 surrogate ewes, and seven lambs were born. Genomic DNA from the lambs was genotyped by PCR, and the transgene could be detected in extra embryonic tissues from five animals. Three of them carried the transgene only in extra embryonic tissues (two of them in placenta and one in umbilical cord). In a forth animal, the transgene could be detected only in liver and placenta. Molecular analysis of the fifth animal revealed transgene presence in almost all evaluated tissues (skin, kidney, muscle, placenta, testis and umbilical cord) (Fig 3A and 3B). However, after primary cell culture of some of the confirmed transgenic tissues (derived from muscle, kidney, lung and placenta), the transgene was only detected in cells derived from umbilical cord.

Southern blotting with a transgene-specific probe was performed on DNA from this animal and showed three bands compatible with integration of the transgene (Fig 3C). In addition, the plasmid coding for Sleeping Beauty 100X transposase could not be detected by PCR, reinforcing the transposase-mediated integration of the transposon (Fig 3D). By cloning and sequencing by splinkerette PCR of one integration site, it was confirmed a specific SB-catalyzed transposition event at the expected TA target dinucleotide site (Fig 3E). The integration site could be mapped to ovine chromosome 23, at the 3’ of olfactory receptor 5D18-like. This genomic integration site was confirmed by PCR on different tissues of the animal, including skin, cryopreserved testis cells and placenta (Fig 3F).

The transgenic lamb obtained by Sleeping Beauty transgenesis died two days after its birth, of a cardiopulmonary arrest. Cells derived from its testis and umbilical cord were cryopreserved.

## Discussion

To our knowledge, this is the first report on the production of transgenic sheep by direct cytoplasmic injection of transposon plasmids into zygotes. The *in vitro* experiments in bovine showed that both Tn5 and Sleeping Beauty transposition systems are capable of resulting in high reporter expression rates in embryos. When both transposition techniques were used for the production of lambs, only Sleeping Beauty transposition resulted in transgenic lambs, with a transgenesis frequency of 29% per born animals.

The limited number of ovine slaughterhouses, in addition to the difficulties to obtain adult sheep ovaries, turned it necessary to make the initial *in vitro* experiments in cattle. Our *in vitro* results in bovine embryos showed no differences in reporter gene expression rates among injection with the transposon alone or in combination with the transposase, for both transposition systems. However, embryo Southern blotting revealed bands compatible with in blastocysts injected with Tn5 transposome complex but not in rette PCR to detect insertion loci in a couple of embryos.ned aintegration of the transposon for the group injected with the Tn5 transposome complex but not in blastocysts injected only with the transposon fragment. In the latest group, only one embryo showed a band, corresponding to transposon concatamers at the blastocyst stage. These results for Tn5 transposon mediated transgenesis in bovine strongly suggest that Tn5 transposase is active in bovine embryos and is responsible for the transposon integration into the genome. In blastocysts injected with plasmids coding for the Sleeping Beauty transposition system, it was not possible to distinguish discrete bands compatible with transgene integration. A strong signal was detected in the analyzed blastocysts and this appears to be related to the episomal presence of the plasmids at the blastocyst stage. It was previously reported Southern blot of embryos injected with SB mRNA and the linear transposon [23,51]. However, to our knowledge, no previous reports exist on the use of embryo Southern blotting on blastocysts injected with plasmids. Our observations here are clear proof of the episomal persistence of plasmid at least, up to the blastocyst stage, when they are injected covalently closed circular (ccc) into the cytoplasm of zygotes. This agrees with previous reports showing that plasmid injection into the cytoplasm of fertilized bovine and murine eggs is a highly efficient and simple alternative for ectopic expression of foreign DNA in embryos [38,52]. Therefore, only the production of live animals could help to address the integration status of the transgene for this technique.

For that reason, we compared Tn5 and Sleeping Beauty systems *in vivo* in ovine. Although embryo Southern blotting showed bands compatible with integration for Tn5 transgenesis in bovine, we could not produce transgenic lambs with this technique. We had speculated that cytoplasmic injection with Tn5 transposome would allow immediate availability of the complex, increasing transposition events early in the embryo and, thus, reducing mosaicism. However, the only born animal produced with this technique showed no transgene signals in the analyzed tissues. In addition, the low pregnancy rates obtained might indicate that the transposon jumps to essential gene coding regions. In any case, it should be necessary to produce more animals in order to determine Tn5 efficiency. Previously, Suganuma et al. [26] reported the production of transgenic mice by Tn5 transposition in combination with ICSI, with 21% efficiency respect to born animals. Although some lambs were obtained by ICSI [53,54], the ICSI technique remains highly inefficient in domestic species, mainly due to improper sperm decondensation [42,55]. Therefore, we decided to adapt the technique to cytoplasmic injection of *in vivo* produced zygotes.

For Sleeping Beauty transgenesis, we were able to produce two transgenic sheep, with efficiencies comparable to previous reports in pigs [23,25]. Regarding the integration status of the transgene, one of the transgenic lambs showed signals of the transposon in almost every analyzed tissue, including cells derived from testis. Southern blot of different tissues of this animal revealed signals compatible with three independent integration sites of the transposon. One of these integration sites could be characterized by splinkerette PCR and confirmed by nested-PCR. The expected TA dinucleotide was found at this site of integration, in agreement with the occurrence of SB-catalyzed transposition. It was previously reported that the majority of SB insertions occur outside of genes [56,57], and the integration site detected in this transgenic lamb agrees with those previous observations. Unfortunately, the transgenic animal died two days after its birth. The second animal obtained was mosaic and the transgene could only be detected in liver and extra-embryonic tissues. An interesting observation for SB transpositional transgenesis was that three additional animals harbored the transgene in extra embryonic tissues (two of them in placenta and one in umbilical cord). It is likely that the higher number of cells allocated to the extra embryonic tissues in early embryo development could be the reason for this observation.

The many advantages that Sleeping Beauty transgenesis encompasses, including its simplicity, high cargo capacity [58], lack of the biosafety concerns observed for lentiviruses, preferential integration of the transgenes into euchromatin and the avoidance of transcriptional silencing [59], turn this technique into a powerful method for transgenic livestock production. The results presented here provide another advantage: its high species adaptability. This report demonstrates the easy and highly efficient production of transgenic bovine embryos and lambs by transposon cytoplasmic injection. In particular, Sleeping Beauty transgenesis mediated by simple cytoplasmic injection of plasmid proved to be efficient for the production of transgenic sheep for pharmaceutical approaches.
